# Mapping under-five child malaria risk that accounts for environmental and climatic factors to aid malaria preventive and control efforts in Ghana: Bayesian geospatial and interactive web-based mapping methods

**DOI:** 10.1186/s12936-022-04409-x

**Published:** 2022-12-15

**Authors:** Justice Moses K. Aheto

**Affiliations:** 1 Department of Biostatistics, School of Public Health, College of Health Sciences, University of Ghana, Accra, Ghana; 2 WorldPop, School of Geography and Environmental Science, University of Southampton, Southampton, SO17 1BJ UK; 3 College of Public Health, University of South Florida, Tampa, FL USA

**Keywords:** Malaria, Under-five malaria, Mapping malaria risk, Bayesian methods, Geospatial methods, Geostatistical methods, Interactive web-based mapping, Predictors, Sub-Saharan Africa

## Abstract

**Background:**

Under-five child malaria is one of the leading causes of morbidity and mortality globally, especially among sub-Saharan African countries like Ghana. In Ghana, malaria is responsible for about 20,000 deaths in children annually of which 25% are those aged < 5 years. To provide opportunities for efficient malaria surveillance and targeted control efforts amidst limited public health resources, the study produced high resolution interactive web-based spatial maps that characterized geographical differences in malaria risk and identified high burden communities.

**Methods:**

This modelling and web-based mapping study utilized data from the 2019 Malaria Indicators Survey (MIS) of the Demographic and Health Survey Program. A novel and advanced Bayesian geospatial modelling and mapping approaches were utilized to examine predictors and geographical differences in under-five malaria. The model was validated via a cross-validation approach. The study produced an interactive web-based visualization map of the malaria risk by mapping the predicted malaria prevalence at both sampled and unsampled locations.

**Results:**

In 2019, 718 (25%) of 2867 under-five children surveyed had malaria. Substantial geographical differences in under-five malaria risk were observed. ITN coverage (log-odds 4.5643, 95% credible interval = 2.4086–6.8874), travel time (log-odds 0.0057, 95% credible interval = 0.0017–0.0099) and aridity (log-odds = 0.0600, credible interval = 0.0079–0.1167) were predictive of under-five malaria in the spatial model. The overall predicted national malaria prevalence was 16.3% (standard error (SE) 8.9%) with a range of 0.7% to 51.4% in the spatial model with covariates and prevalence of 28.0% (SE 13.9%) with a range of 2.4 to 67.2% in the spatial model without covariates. Residing in parts of Central and Bono East regions was associated with the highest risk of under-five malaria after adjusting for the selected covariates.

**Conclusion:**

The high-resolution interactive web-based predictive maps can be used as an effective tool in the identification of communities that require urgent and targeted interventions by programme managers and implementers. This is key as part of an overall strategy in reducing the under-five malaria burden and its associated morbidity and mortality in a country with limited public health resources where universal intervention is practically impossible.

**Supplementary Information:**

The online version contains supplementary material available at 10.1186/s12936-022-04409-x.

## Background

Malaria is a deadly disease and remains one of the severe global public health and development challenge particularly in sub-Saharan Africa (SSA) where under-five malaria infection is the leading cause of under-five mortality (U5M) due to their vulnerability. It is the leading cause of illness and deaths in most of the malaria affected countries where young children and pregnant women are the most affected groups. Notably, malaria is considered entrenched global health problem and Ghana is of no exception due to its significant number of deaths associated with the disease in the country [1, 2]. Malaria is also a driver of low productivity and poverty on the individuals and exerts financial burden on families and the economy [3]. An estimated number of 405,000 persons died due to malaria infections out of which majority were young children from SSA in 2018 as against the 2017 figure of 416,000 deaths and 585.000 deaths in 2010. In 2016, there were an estimated number of 216 million clinical episodes caused by malaria, an increase of 5 million over the previous year [2, 4, 5]. Despite the global rapid malaria control efforts that led to malaria mortality reduction by 25% from the year 2010 to 2016, the malaria prevalence and mortality rates remain high in SSA countries where 14 out of 15 countries in SSA accounted for 80% of the world malaria burden with national and sub-national differences [2, 4, 6].

In 2018, the most vulnerable group hardest hit by malaria are children under-five who accounted for 67% of all malaria deaths globally [5]. SSA had the highest burden of malaria where about 90% of all malaria deaths occur with children under-five accounting for about 78% of these deaths [7]. This link between malaria and under-five deaths also poses a great danger to achieving the Sustainable Development Goals (SDG) 3 target 2.1 because the U5M rates are among the health indicators of utmost importance globally. It is the goal 3 target 2.1 of the SDGs that is expected to be reduced to internationally agreed targets of at least 25 per 1,000 livebirths by 2030 [8], but several countries especially those in SSA like Ghana are struggling to meet this target [9, 10]. Thus, addressing the problem of under-five malaria will be beneficial to the global fight against U5M.

Ghana was among the 10 highest burden countries in Africa in 2018 that reported the highest increase in malaria cases compared to the previous year [5], where about 20,000 children die annually of which 25% are those aged < 5 years [7]. In Ghana, malaria is considered endemic in all the regions with national prevalence of 14% in 2019 against the previous prevalence of 21% and 27% in 2016 and 2014, respectively, among under-five children. However, marked regional geographic disparities exist in the under-five malaria prevalence in Ghana with the highest prevalence recorded in the Western (27%) and lowest recorded in the Greater Accra (2%) regions. Thus, the under-five malaria prevalence across the country varied [11], demonstrating the need for examining more localized spatial trends in malaria. Unfortunately, information on localized spatial distributions and predictors of under-five malaria supported with web-based mapping, which are critical for effective design of intervention strategies that will enhance the survival of under-five children amidst available limited public health resources are not readily available.

At the global level, the United States President’s Malaria Initiative (PMI) launched in 2005 led to an increased availability of insecticide-treated nets (ITNs), anti-malarial treatment and rapid diagnostic tests and indoor residual spraying, which led to a significant reduction in under-five mortality in SSA [12]. The success of the “*for a malaria-free world 2008–2015* initiative”, the Roll Back Malaria Partnership outlined an action plan dubbed, “Action and Investment to Defeat Malaria (AIM) 2016–2030” [13]. In May 2015, the *Global technical strategy for malaria (GTSM) 2016–2030* which sets the target of reducing global malaria incidence and mortality rates by at least 90% by 2030 was adopted by the World Health Assembly. The strategy was updated in the year 2021 to reflect the lessons learned in the global malaria response between 2016 to 2020. It provides a comprehensive framework to guide nations in their efforts to fast-track progress towards elimination of malaria by emphasizing the need for universal coverage of core malaria interventions for all populations at risk. At the heart of the strategy is the utmost need to use high-quality surveillance data for decision-making [14]. The alignment of the timeframe of the vision of AIM and GTSM to that of the SDG underscores the need to address the problem of under-five malaria to ensure the realization of SDG goal 3. Nonetheless, under-five malaria continues to be a significant cause of childhood deaths in SSA which militates against the progress towards the achievement of the sustainable development Goal 3 target 2.1.

In Ghana, despite several national policies and interventions (e.g. Community-based Health Planning and Services (CHPS), Child Health Policy 2007–2015 and National Health Insurance, 2014–2020 Ghana Strategic Plan for Malaria Control) [1, 10, 15] rollout to improve and promote health of children, the under-five malaria and its resultant under-five mortality rates remain high in the country. The focus of the 2014–2020 Ghana Strategic Plan for Malaria Control is to scale up preventive interventions to reduce the malaria morbidity and mortality burden by 75% by the year 2020 [16].

There is limited knowledge on localized geospatial distribution of under-five malaria risk and how certain environmental predictors could help explain geographical differences in under-five malaria risk in Ghana. Also, malaria burden is a continuous phenomenon that requires high-quality surveillance data and constant surveillance to inform malaria control strategies.

This study, therefore, attempts to fill these gaps. The study aims to estimate, predict, and map localized under-five malaria risk using novel Bayesian geospatial modelling approaches while adjusting for critical environmental factors, with the goal of identifying communities at high-risk of malaria burden where control efforts, interventions, and further research can be targeted to address the problem of under-five malaria and its associated morbidity and mortality.

## Methods

### Setting, design and sample

Ghana is in West Africa and covers a total area of 238,538 km^2^. It lies between latitude 4  and 12 N and longitudes 4 W and 2 E. It is bordered in the south by the Gulf of Guinea, Côte d’Ivoire to the west, Togo to the east, and Burkina Faso to the north. Presently, Ghana has 16 administrative regions.

Data from the 2019 GMIS of the DHS program was used in this study [11]. The 2019 GMIS is the second round of the survey with the first round conducted in 2016 which provides a population-based estimates of malaria indicators as a supplement to the routine administrative data collected in the country that are used to inform strategic planning and evaluation of the Ghana Malaria Control Programme [11]. Computer-assisted personal interviewing (CAPI) was employed to collect the data. In the survey, information on malaria prevention, treatment, and prevalence is collected. The data is freely available online at DHS MEASURE Program website [17]. Parents or guardians consent were sort for children aged 6–59 months who were tested for anaemia and malaria infection. The study used a biomarker questionnaire to record the results of the anaemia and malaria testing of the children aged 6–59 months. This study used data on under-five children from the biomarker dataset which has malaria RDT results on 2867 under-five children residing in 192 geographical locations (clusters). Detailed description of the survey methods employed in the 2019 GMIS is available elsewhere [11].

The Ghana Malaria Indicator Survey (GMIS) is based on a two-stage sampling design. The sampling was based on ten administrative regions. Each region was divided into urban and rural areas, resulting in twenty sampling strata. Enumeration areas (EAs) were sampled from each stratum. In the first stage, 200 EAs (97 in urban areas and 103 in rural areas) were selected with probability proportional to EA size. In the second stage of selection, approximately 30 households were selected from each cluster to make up a total sample size of 6,002 households of which 5388 were occupied at the time of field work. A total of 5799 household were interviewed among the occupied households, resulting in 99.4% response rate. Of the 5246 eligible women, about 5181 women aged 15–49 years (representing 98.8% response rate) who were either permanent residents of the selected households or visitors who stayed in the household the night before the survey were interviewed. All children aged 6–59 months from the interviewed households were eligible for malaria testing upon parental or guardian consent [11].

### Outcome variable

The outcome variable of interest is the number of under-five children with positive test on rapid diagnostic test (RDT) kit in each sampled cluster. The RDT malaria test was conducted by taking a drop of blood with the SD BIOLINE Malaria Ag Pf RDT and tests for one antigen, histidine-rich protein II (HRP-II), specific to *Plasmodium falciparum*, the major cause of malaria in Ghana. The RDT kit produces result in 15 min [11].

### Covariates

Though the main goal of the study is to predict and map under-five malaria risk, the study adjusted for selected environmental factors to allow for examination of how these factors help explain some of the spatial variability in under-five malaria risk across Ghana. These factors include insecticide-treated nets (ITNs) coverage (i.e., proportion of the population protected by ITNs), travel time (time required to reach a high-density urban centre), aridity (ranging from most arid to most wet), enhanced vegetation (ranging from least vegetation to most vegetation), annual temperature (mean temperature), and precipitation (average precipitation–per month). A detailed description of the methods and procedures employed to generate these geospatial covariates and their sources are published elsewhere [18]. The consideration of these environmental and climatic covariates was based on the available literature on predictors of malaria and other health outcomes [19–22]. Following recommended strategy [20, 23], the study accounted for the displacement of the GPS coordinates of the sampled cluster locations by creating 2 km buffers for urban and 5 km buffer for rural settings to ensure that the correct cluster centroids were captured in the analysis.

### Geospatial analysis

### Model formulation

Following a previous modelling approach [20], we employed a Bayesian Geospatial model [20, 24] to study spatial risk in under-five malaria while adjusting for environmental predictors. Consider ${Y}_{i}$ to be the number of under-five children with positive RDT test out of the total ${N}_{i}$ under-five children sampled per geographical cluster. Given the true malaria risk $P\left({z}_{i}\right)$ at location ${z}_{i}$, the number of under-five children with positive RDT test out of the total number of under-five children sampled follows a binomial distribution formulated as:

$${Y}_{i}|P\left({z}_{i}\right) \sim Binomial\left({N}_{i}, P\left({z}_{i}\right)\right),$$

$$logit\left(P\left({z}_{i}\right)\right)= {\beta }_{0}+{\varvec{d}}{\left({{\varvec{x}}}_{{\varvec{i}}}\right)}^{\mathbf{^{\prime}}}\beta +S\left({z}_{i}\right).$$

where ${\beta }_{0}$ is the intercept parameter which by default is assigned Gaussian prior with mean and precision to be zero (0), $d\left(.\right)$ is a vector of observed environmental predictors of the outcome variable $Y$, $\beta$ is a vector of spatial regression coefficients for the covariates which by default was assigned Gaussian prior with mean zero (0) and precision 0.001, and $S\left(.\right)$ is a spatially structured random effect and follows a zero-mean Gaussian process with variance ${\sigma }^{2}$ and a given correlation function

$$\rho \left(u\right)=corr\left\{S\left({z}_{i}\right), S({z}_{j})\right\}$$

where $u$ is the Euclidean distance between locations ${z}_{i}$ and ${z}_{j}$. There are various parametric families for $\rho \left(u\right)$ as outlined by Diggle (2007) [25]. In the current analysis, the study use the Matérn class of covariance function[26] given by

$$Cov\left(S\left({z}_{i}\right),S\left({z}_{j}\right)\right)=\frac{{\sigma }^{2}}{{2}^{v-1}\Gamma \left(v\right)}{\left(k||{z}_{i}-{z}_{j}||\right)}^{v}{K}_{v}\left(k||{z}_{i}-{z}_{j}||\right).$$

Here, ||. || denotes Euclidean distance, ${\sigma }^{2}$ represents the spatial variance, $v$ is the shape parameter which determines the smoothness of$S\left(z\right)$, in the sense that $S\left(z\right)$ is $v-1$ times mean-square differentiable and the scale parameter $\kappa >0$ is related to the practical range $\rho =\frac{\sqrt{8v}}{k}$, the distance at which the spatial correlation approaches 0.1 or is negligible, ${\kappa }_{v}(.)$ is the modified Bessel function of second kind and order$v>0$.

The model was implemented under the Integrated Nested Laplace Approximation (INLA) approach [27] with Stochastic Partial Differential Equation (SPDE) strategy [28]. Based on a previous study [20], a mesh for inference and prediction was created for the SPDE strategy because the data (i.e., geostatistical data) points in this study do not have explicit neighbours required by the SPDE strategy unlike areal data. The description of the mesh creation is provided in Additional file 1. The detailed procedures for mess creation are published elsewhere [20, 29].

In this study, nine (9) models were set up: two (2) non-spatial models with different set of covariates included, one spatial model without covariates, and five (5) spatial models with different set of covariates. The Watanabe-Akaike information criterion (WAIC) was employed to investigate how well each of these nine (9) models fits the data, and to select the model that relatively fits the data well among the competing models. The level of uncertainty in the fitted model estimates were quantified by estimating the 95% credible intervals and the standard errors and map these uncertainties continuously across the whole of Ghana. Furthermore, the study compares the predictive maps for the spatial model with covariate and spatial model without covariates to examine if the included covariates in the spatial model explained some differences in malaria prevalence predictive maps. The study investigated how well the predictive model performs in the presence of new data via cross-validation procedure by splitting the data into training and validation sets, a common and generally accepted model validation approach in this area [30]. The R-INLA package [29, 31] was used for all the analyses.

### Model validation

It is critical to examine how well the predictive model performs, especially in the presence of new data. This study employed cross-validation approach to assess the predictive performance of the model under out of sample procedure. First, the data was split into training and validation sets, and set a seed of 123 to make the partition reproducible. The model was trained on 75% of the samples and tested on 25% of the samples. The study assessed the model predictive performance by plotting the observed and the predicted malaria prevalence and estimated the resultant correlation.

### Interactive web-based mapping of the predicted malaria prevalence

To support policymakers with readily available quality data for targeted policy and intervention strategies, especially for malaria surveillance amidst limited public health resources in these settings, the study produced interactive web-based maps for the predicted malaria prevalence to improve visualization and identification of higher risk communities for urgent intervention and further research in this setting where universal intervention is practically impossible due to limited public health resources. The *spatsurv*, *rgdal*, *leaflet*, and *sp* packages in *R* version 4.2.0 and *RStudio* [32, 33] were used to support the development of the interactive web-based predicted malaria prevalence maps.

### Ethical consideration

Permission was granted by DHS MEASURE Program to use the 2019 GMIS data for the study. The data is freely available after a simple, registration-access request at the link https://dhsprogram.com/data/dataset_admin/index.cfm. The protocol for the 2019 GMIS was approved by the Ghana Health Service Ethical Review Committee and ICF’s Institutional Review Board [11].

### The role of the funding source

The present study did not receive any support from any funding source. Also, the funders of the original survey played no role in the design, data collection, analysis, interpretation, writing of the manuscript, and the decision to submit this manuscript. The author confirm that he has full access to all the data in this study and accept responsibility to submit for publication.

## Results

The study analyzed data on 2867 children aged below 5 years residing in 192 clusters (communities). A total of 718 (25%) children under-five had malaria in the study in 2019.

### Model selection results

To select a good model among the competing models we fitted to predict the malaria prevalence, this study employed the Watanabe-Akaike information criterion (WAIC). The model with the smallest WAIC value is preferred. In all, nine (9) competing models were fitted and Model 6 which contained aridity, ITN coverage, and travel time had the smallest WAIC value (WAIC = 689.79) compared to all other models fitted, an indication of a better model fit for the study (Table 1). Thus, the study present and discuss the results based on the full spatial model (i.e., Model 6, Table 1) presented in Table 2.

**Table 1:** Model selection for the fitted Bayesian Geospatial models

Parameters	WAIC
Full non-spatial model	
Model 1: Aridity, ITN coverage, Travel times, Precipitation, Vegetation, Temperature	903.38
Model 2: Aridity, ITN coverage, Travel times	905.18
Spatial models	
Model 3: Null spatial model	698.39
Model 4: Aridity	696.63
Model 5: Aridity, ITN coverage	692.34
Model 6: Aridity, ITN coverage, Travel times	689.79
Model 7: Aridity, ITN coverage, Travel times, Precipitation	692.00
Model 8: Aridity, ITN coverage, Travel times, Precipitation, Vegetation	690.64
Model 9: Aridity, ITN coverage, Travel times, Precipitation, Vegetation, Temperature	691.91

*WAIC* Watanabe-Akaike information criterion

Lower values of the WAIC indicate better model fit

**Table 2:** Predictors of malaria prevalence in the non-spatial and spatial Bayesian models

Parameter	Mean log odds (95% Credible intervals)
Full non-spatial model	
Intercept	− 4.9129 (− 5.7024, − 4.1390)
ITN Coverage	4.0385 (3.1535, 4.9407)
Travel time to health facility	0.0037 (0.0020, 0.0054)
Aridity	0.0476 (0.0271, 0.0681)
Spatial model	
Null spatial model	
Intercept	− 1.1943 (− 1.4532, − 0.9415)
${\sigma }^{2}$(spatial variance)	1.3218 (0.8211, 1.8869)
Range nominal	0.2738 (0.1365, 0.4271)
$\kappa$(kappa)	11.1711 (5.6585, 17.6613)
Full spatial model	
Intercept	− 2.9184 (− 4.0083, − 1.9530)
ITN Coverage	4.5643 (2.4086, 6.8874)
Travel time to health facility	0.0057 (0.0017, 0.0099)
Aridity	0.0600 (0.0079, 0.1167)
${\sigma }^{2}$(spatial variance)	0.8772 (0.5061, 1.2915)
Range nominal	0.2917 (0.1250, 0.4886)
$\kappa$(kappa)	10.8059 (4.6372, 18.1236)

### Predictors of malaria prevalence from the Bayesian spatial models

**Fig. 1:**
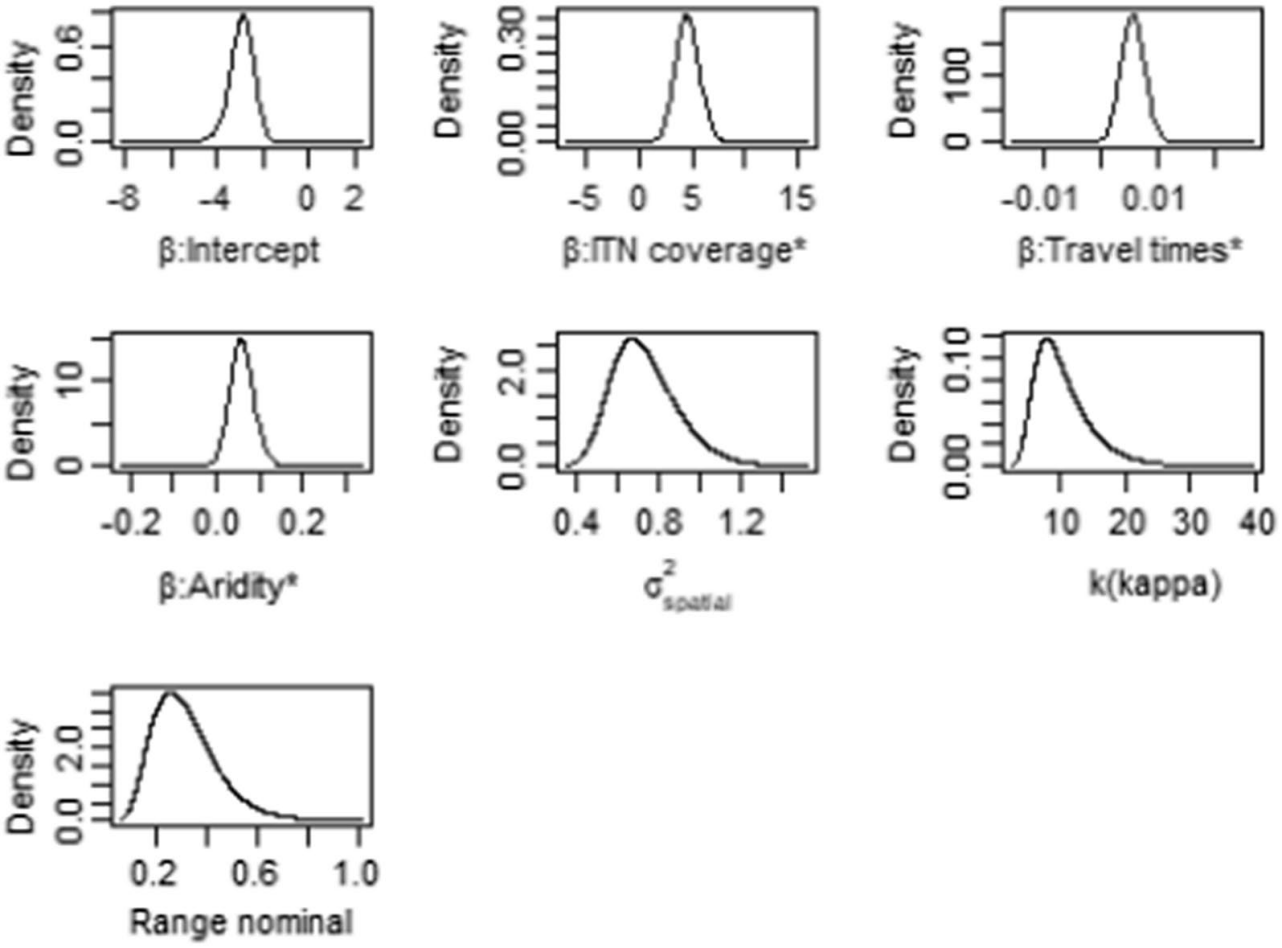
Posterior distribution of the effect of the predictors of malaria prevalence and the hyper parameters in the Bayesian spatial model in 2019 among under-five children in Ghana. *Denotes significant covariates

The study presents the results based on the full spatial model (Model 6) in Table 2 and Fig. 1. ITN coverage (log-odds 4.5643, 95% credible interval = 2.4086–6.8874), travel time (log-odds 0.0057, 95% credible interval = 0.0017–0.0099) and aridity (log-odds = 0.0600, credible interval = 0.0079–0.1167) were found to be predictive of under-five malaria risk. The estimated spatial variance (${\sigma }^{2}$) is 0.8772 (95% credible interval = 0.5061–1.2915) and the estimated range is 0.2917 (95% credible interval = 0.1250–0.4886) while the kappa ($\kappa$) is 10.8059 (95% credible interval = 4.6372–18.1236) (Table 2).

Figure 1 shows the posterior (marginal) distributions of the fixed and random (hyper) parameters of the Bayesian Geospatial model (i.e., full spatial model—Model 6) presented in Table 2, which provides a fuller understanding of the posterior distributions of the model parameters and the appropriate quantification of uncertainty around the estimates unlike the frequentist approaches.

### Geospatial analysis and interactive web-based mapping

**Fig. 2:**
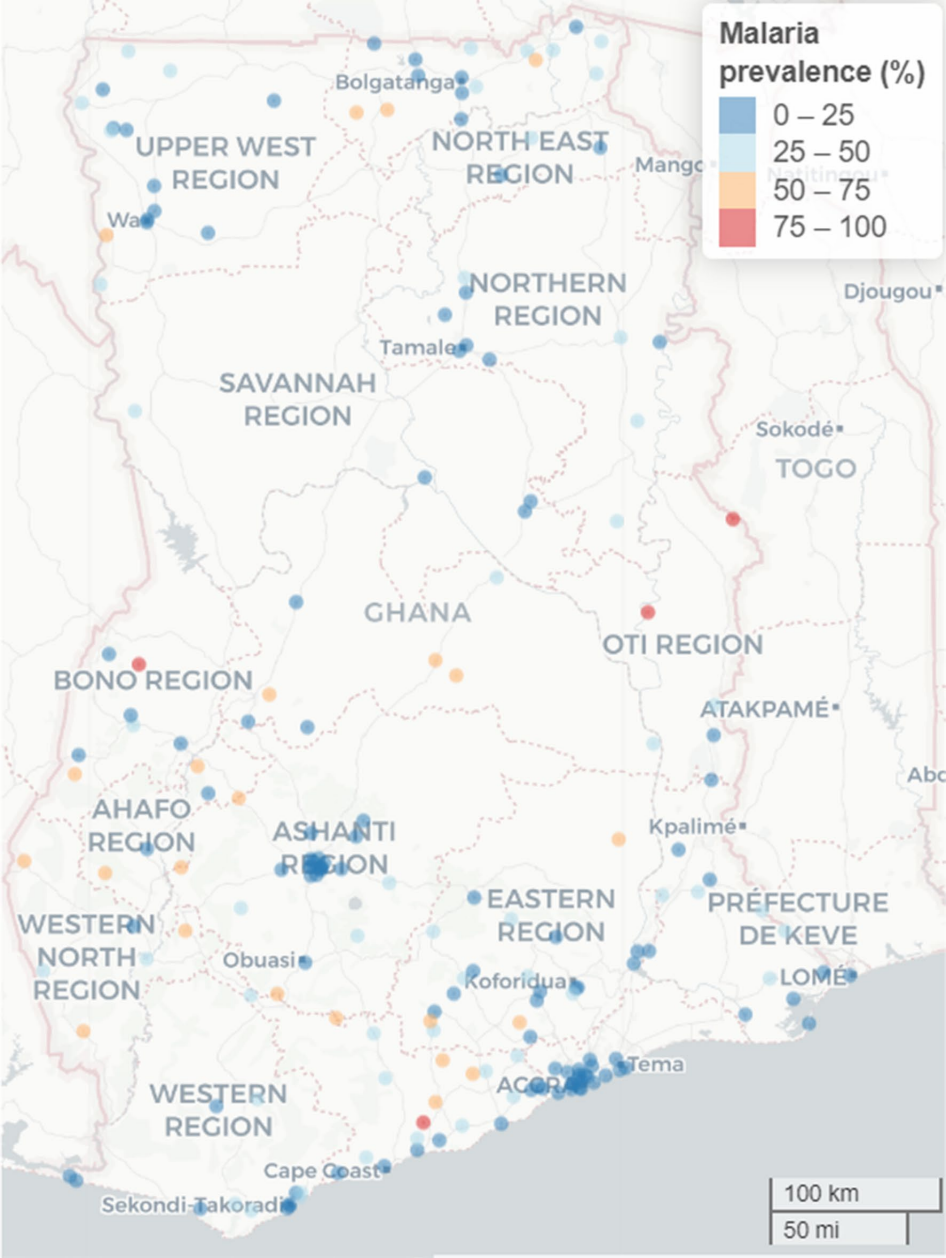
Empirical (observed) malaria prevalence in study locations in Ghana, 2019. Each circle represents a study location

This study analyzed data on children residing in 192 communities which are geographically indexed. In Fig. 2, we showed the location (centroid of clusters) of communities used in this study and their respective empirical (observed) malaria prevalence (coloured) at the sampled locations. Communities with red highlighted circles had observed malaria prevalence of between 75 to 100% while those with blue highlighted had observed 0–25% prevalence.

### Model validation results

**Fig. 3:**
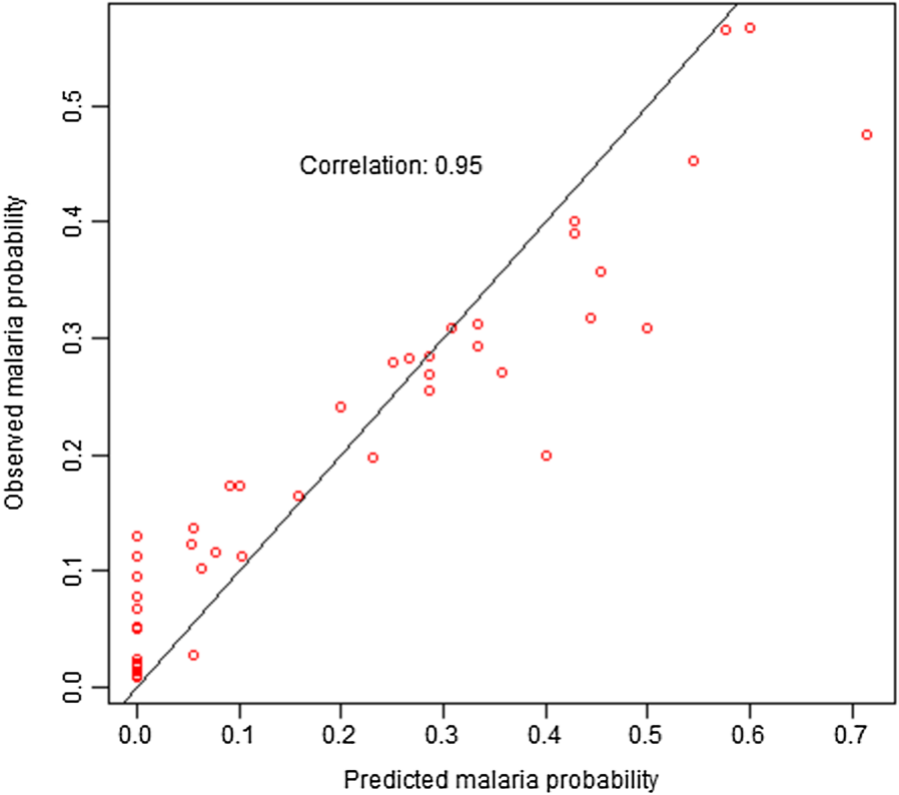
Model validation for the final Bayesian geospatial model for predicting malaria prevalence among children under-five in 2019 in Ghana

Presented in Fig. 3 is the model validation results to determine the predictive ability of the final model, especially in the presence of new data. Given the high correlation of 0.95, the fitted Bayesian geospatial prediction model is very good for predicting malaria prevalence spatially.

**Fig. 4:**
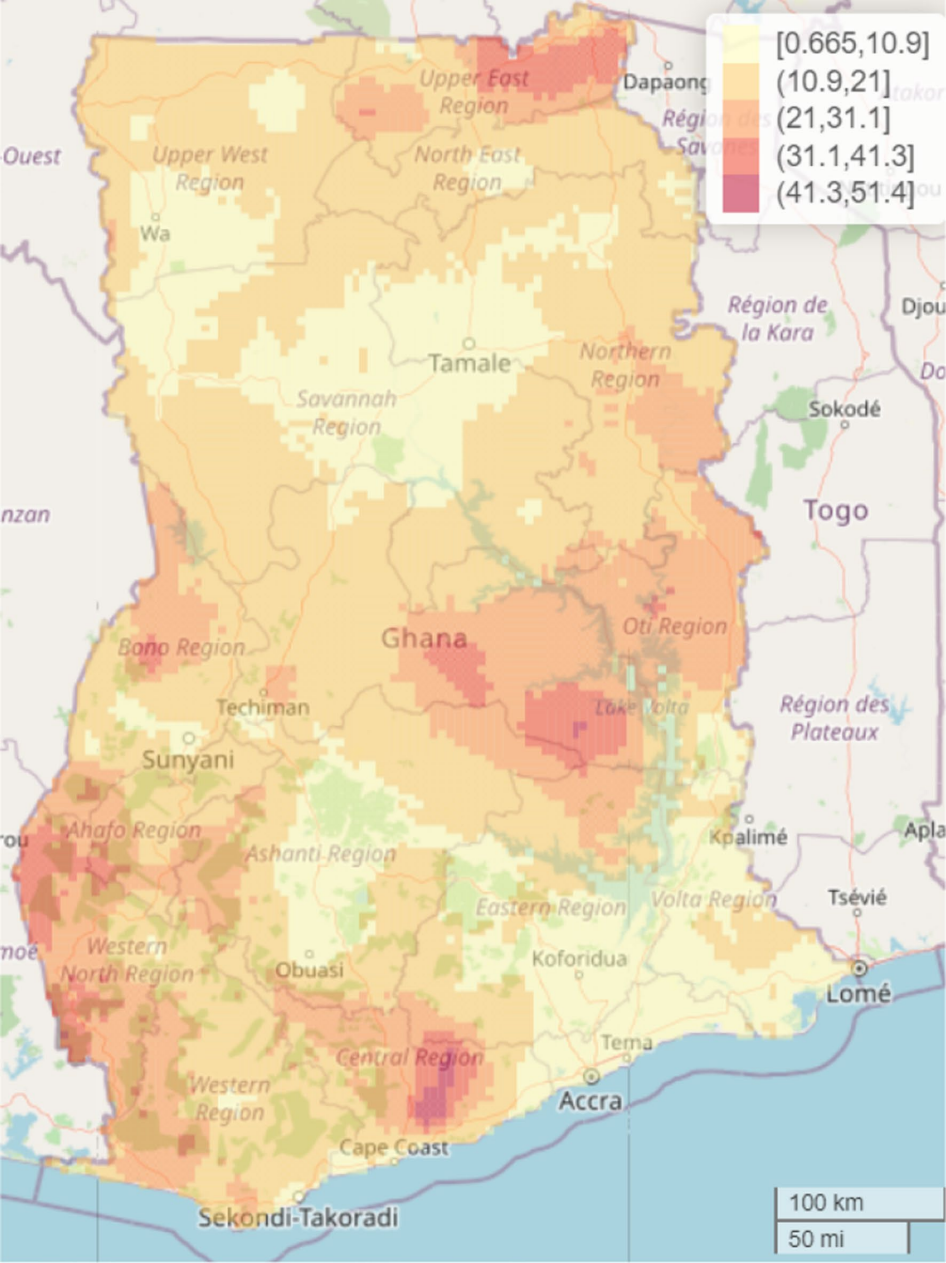
Predicted malaria prevalence in 2019 among under-five children in Ghana. The interactive web-based version of this map can be found online

**Fig. 5:**
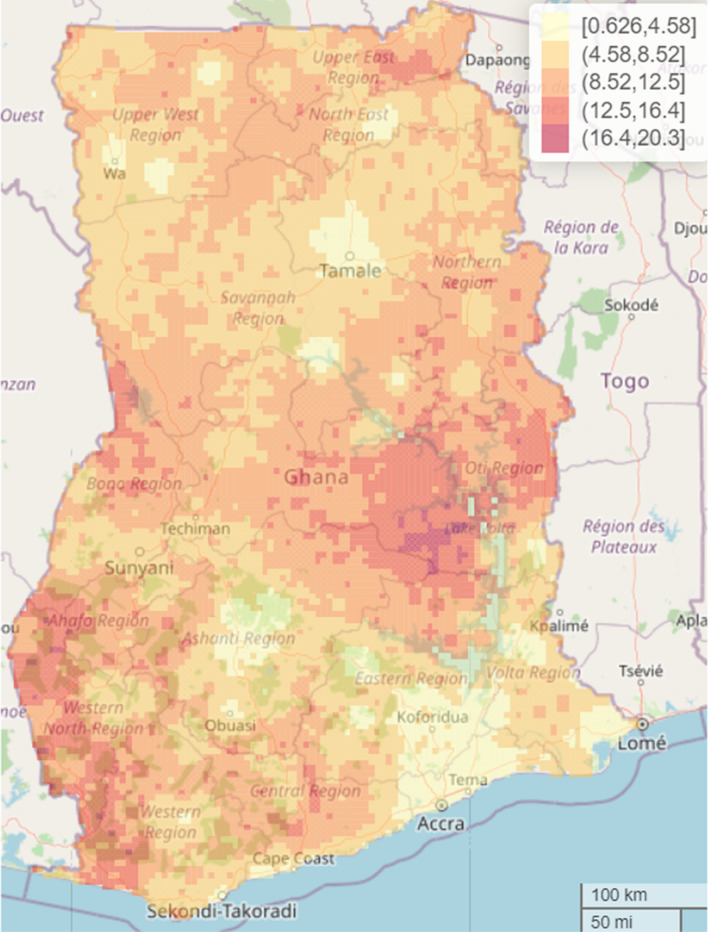
SEs of predicted malaria prevalence in 2019 among under-five children in Ghana. The interactive web-based version of this map can be found online

The study found significant geographical differences in the predicted malaria prevalence in the country. The overall predicted malaria prevalence was 16.3% (standard error (SE) 8.9%) with a range of 0.7% to 51.4% in the spatial model with covariates and overall prevalence of 28.0% (SE 13.9%) with a range of 2.4 to 67.2% in the spatial model without covariates. Thus, inclusion of the covariates contributed to explaining some of the geographical differences found in the predicted malaria prevalence. Here, the focus is on the interpretation of the results from the spatial model that included the covariates. Residing in parts of Central (> 41.3 to 51.4%), Bono East (> 41.3 to 51.4%) and Upper East (> 31.1 to 41.3%) regions was associated with highest risk of under-five malaria after adjusting for the selected covariates. Other relatively high-risk regions include Upper East, Oti, Bono, Ahafo and Western North that recoded a prevalence of > 31.1 to 41.3% whereas parts of Greater Accra, Eastern, Northern, Volta, Upper West, Savannah, and Ashanti regions showed some of the lowest prevalence of 0.7 to 10.9% (Fig. 4). The interactive web-based version of Fig. 4 can be found in Additional file 2: Fig. S1. The standard errors (SEs) were presented in Fig. 5 to quantify the uncertainty associated with our estimates presented in Fig. 4. The interactive web-based version of Fig. 5 can be found in Additional file 2: Fig. S2. The estimated mean SEs is 8.9% with a range of 0.67 to 20.3%, suggesting low level of uncertainty for the estimates, hence reliable estimates.

**Fig. 6:**
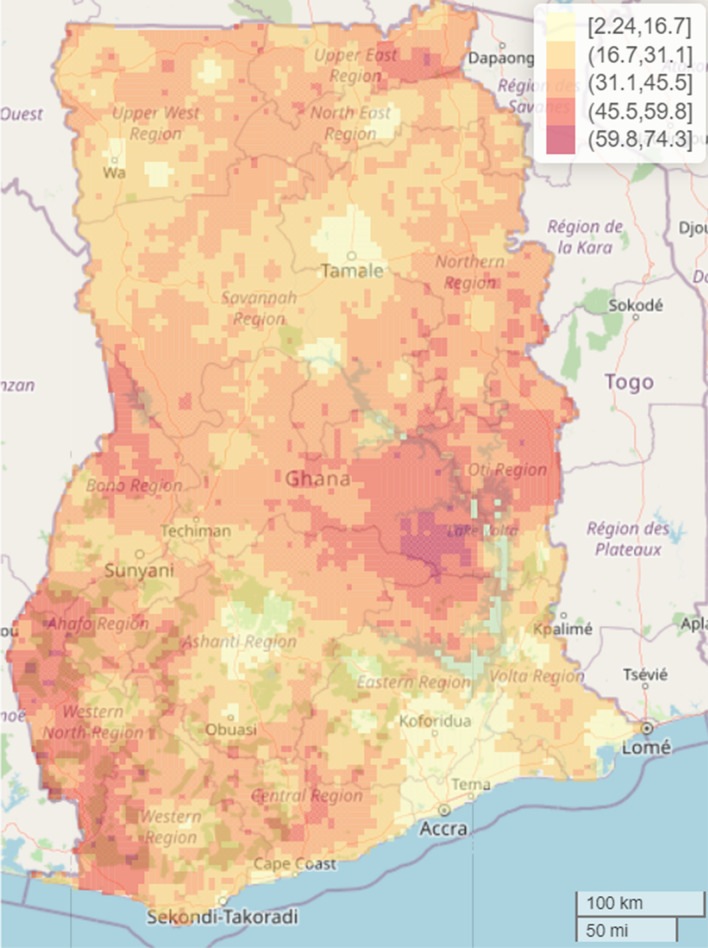
Predicted width of the 95% credible intervals of malaria prevalence in 2019 among under-five children in Ghana. The interactive web-based version of this map can be found online

Presented in Fig. 6 is the width of the 95% credible interval for the predicted malaria prevalence presented in Fig. 4, and the interactive web-based version of Fig. 6 can be found in Additional file 2: Fig. S3. The width of the 95% credible interval ranges from 2.2 to 74.3% with the highest observed in parts of Bono East (59.8 to 74.3%) and the lowest in parts of Greater Accra (2.2 to 16.7%) regions.

### Comparing spatial model with covariate and spatial model without covariates

**Fig. 7:**
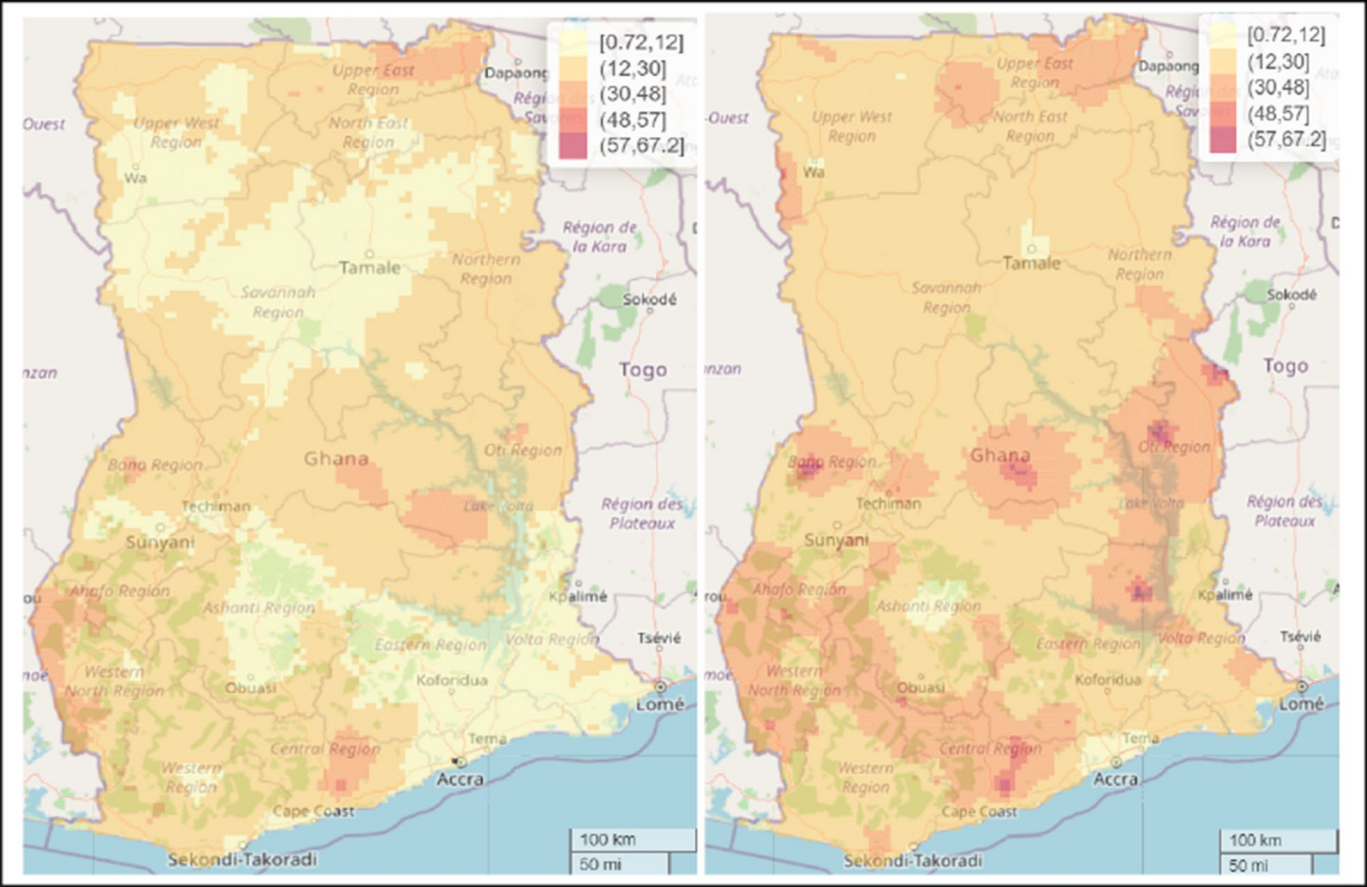
Comparing the predictive maps for spatial model with covariate (left panel) and spatial model without covariates (right panel) from the Bayesian Geospatial models for malaria prevalence in 2019 among under-five children in Ghana

To permit better comparison and understanding between the malaria predictive maps, we fixed the scale for both the predictive maps for the spatial model with covariate and spatial model without covariates. The results showed that the inclusion of these covariates helped explain some of the differences in malaria prevalence across the whole of Ghana, especially in the Central, Bono East, Oti, and Bono regions (Fig. 7).

**Fig. 8:**
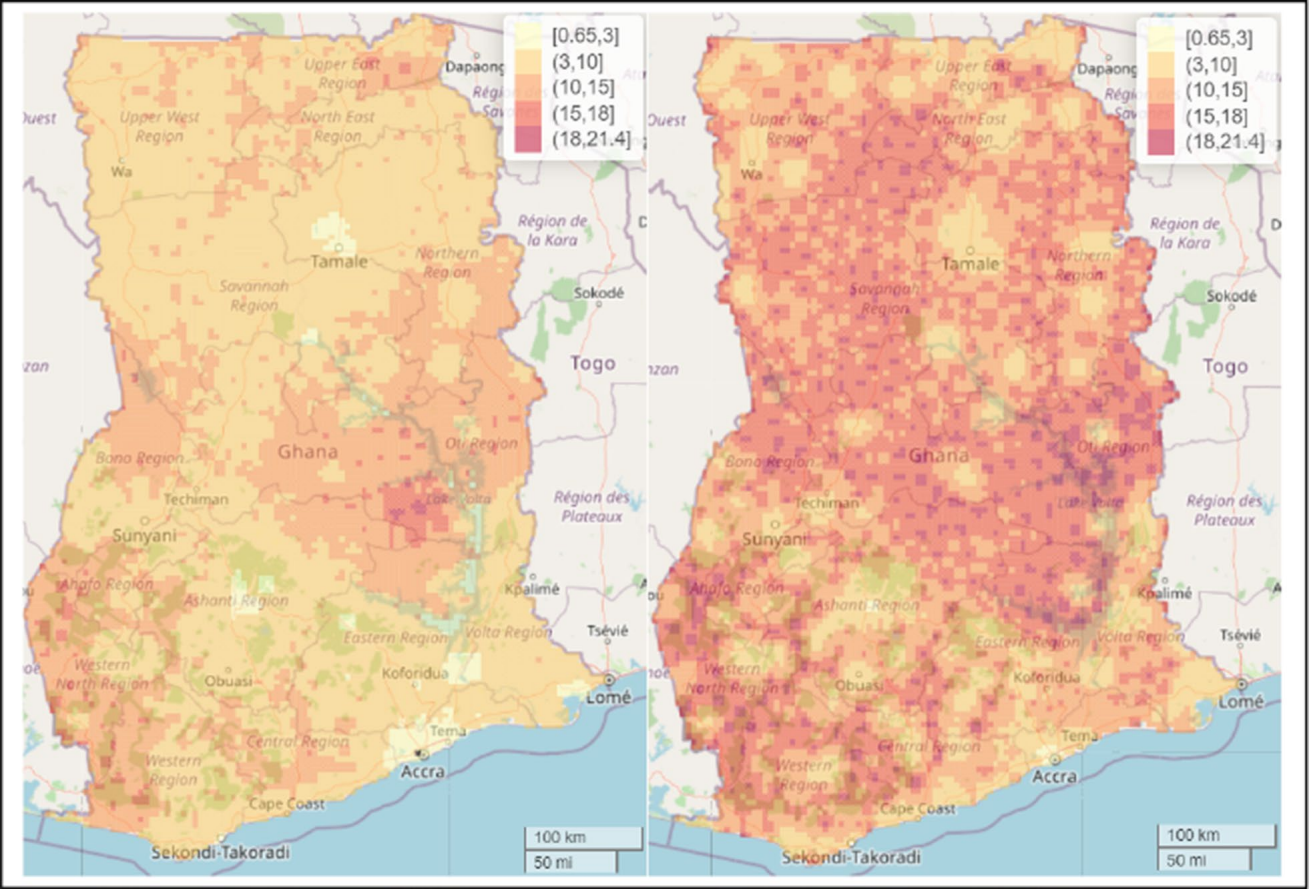
Comparing the level of uncertainty between maps for spatial model with covariate (left panel) and spatial model without covariates (right panel) from the Bayesian Geospatial models for malaria prevalence in 2019 among under-five children in Ghana

We presented the SEs for the estimates presented in Fig. 7 to examine the level of precision of the estimates for the two models. We observed a lower level of uncertainty (i.e., better precision) for the estimates in the spatial model with covariates compared to the spatial model without covariates (Fig. 8). The mean SE associated with the spatial model with covariates was 8.9% compared to the SE of 13.9% for the spatial model without covariates.

## Discussion

This study utilized novel and advanced Bayesian Geospatial models which is often the preferred approach to disease mapping [30] to characterize under-five malaria risk spatially in this study. The need to link health outcomes like malaria to residential location of people is of utmost importance globally and is increasingly being recognized by the international health community and development partners for disease surveillance, monitoring, and control efforts [6, 20, 30, 34–36]. Under-five malaria is among the leading causes of under-five mortality in sub-Saharan Africa. Malaria monitoring and control programmes could heavily benefit from timely, relevant, and accurate high resolution predictive malaria prevalence maps at a more localized levels supported with interactive web-based mapping tools that identify communities with highest burden of malaria risk to inform optimal preventive and targeted control efforts aimed at reducing malaria related morbidity and mortality, especially in settings where universal intervention is practically impossible due to limited public health resources.

Of particular interest in this study is quantification of geographical differences in under-five malaria risk continuously over the whole of Ghana as indicated in the predicted spatial maps. The 5 × 5 km high resolution predictive maps showed substantial geographical differences in the predicted malaria prevalence and identified specific communities/towns with highest concentration of malaria risk, supporting previous studies that observed that health outcomes like malaria, malnutrition, mortality and other related health outcomes exhibit spatial patterns and that the identification of these geographical patterns are of outmost importance urgent and targeted public health policy and intervention, especially in prevention and control efforts with the goal of improving health outcomes in populations at sub-national, national and global levels [6, 7, 20, 35, 37–39]. The overall predicted national malaria prevalence is 16.3% (SE = 8.9%), characterized by substantial localized geographical differences with the highest observed in parts of Central and Bono East regions (41.3–51.4%) and lowest in parts of Greater Accra, Eastern, Northern, Volta, Upper West, Savannah, and Ashanti regions (0.665–10.9%). The findings provide critical information to malaria control programme managers and other stakeholders in public health for urgent and targeted malaria preventive and control efforts, where universal intervention is practically impossible amidst limited public health resources.

Comparing the predictive maps of the estimates of our spatial model which included covariates and the one without covariates, this study found that inclusion of the covariates helped explain some of the geographical differences in malaria risk, and with better accuracy compared to the spatial model without covariates, suggesting the need for researchers in this field to account for environmental and climatic factors that might help explain the malaria risk in this population of children for targeted preventive and control efforts.

To improve visualization, understanding and targeting of scarce available resources to those communities who needed it most (i.e., children highest malaria burden areas), it is recommended that the programme managers and readers use the interactive web-based versions of the predicted maps published online (see figure titles for URL) where they can zoom-in or zoom out on specific towns or communities where the predicted malaria risk is highest or lowest. Generally, the level of uncertainties associated with our estimates are low, suggestive of reasonably accurate estimates. Cross-validation was performed to examine how well our model performs on a new data. The results show a very high correlation of 95%, suggesting that the model is good for correctly predicting malaria risk spatially in this population of children.

The study found environmental and climatic factors like ITN coverage, travel times and aridity to be positively predictive of under-five malaria prevalence. Increase in aridity index ranging from most arid to most wet increases the risk of malaria infection [40], while increase in travel times to reach a high-density urban centre was associated with increased risk of malaria infection, and both findings are in the expected direction. Unexpectedly, increase in ITN coverage, which is increase in proportion of population protected by ITNs was associated with increased risks of malaria infection. This could be due to effect of suppressor variable and/or undetected multicollinearity [41].

The key strength is the ability of the modelling approach to borrow information from sampled locations to create predictions and interactive web-based spatial maps for both the sampled and the unsampled locations in the study over the whole of Ghana, while simultaneously adjusting for environmental and climatic factors. This study accounted for the displacement of the cluster locations, which is typical of the DHS data which ensures that similar approach can be applied accurately on other countries participating in the DHS program. Also, the findings are relevant to the wider population of Ghanaian children and similar populations elsewhere due to the nationwide coverage and representativeness of the survey at the national level. Just like any other study, this study is subject to some limitations so the results should be interpreted with caution: data on spatially referenced malaria data on policy and interventions, distance to the nearest water bodies, type of housing which might explain some of the geographical differences in malaria risk observed were unavailable to be included in the models.

The findings from the present study provide a critical tool for malaria surveillance and monitoring and assessing progress in the fight against malaria and served as an evidenced base for malaria control programme managers and other stakeholders in public health to direct their resources to communities at utmost need, especially in countries like Ghana where available public health resources are very limited, making it practically impossible to rollout a universal intervention. Unlike national, regional or district level estimates that masked real localized differences in risk levels (i.e., ecological fallacy), the modelling and mapping approach enabled more localized evaluation of malaria risk as a continuous phenomenon on finer scales (both at sampled and unsampled locations) over the whole of Ghana, allowing for effective and efficient allocation of the limited available public health resources dedicated to malaria prevention and controls efforts to communities at greatest need. Thus, this study contributes to better understanding of the issue of under-five malaria burden in Ghana.

## Conclusion

The study investigates, model, predict, and presents predictive maps of geographical differences in under-five malaria risk over Ghana. The Bayesian Geospatial modelling of the environmental, and climatic predictors of malaria prevalence and interactive web-based spatial predictive maps provided in this study could be beneficial as an effective tool for the Ghana Health Service and her partners in the development of frameworks to mitigate malaria burden. This study identified communities at highest risk of malaria that may require urgent and targeted interventions and further research amidst limited public health resources in this and other similar settings by public health officers, program managers and implementers, especially where it practically impossible to rollout a universal intervention. The modelling and spatial mapping approaches are critical as part of an overall strategy in reducing the malaria burden amidst limited public health resources available in the country because they can promote effective and sustainable malaria public health programs among under-five children in the country and other similar countries. To answer as-yet unanswered questions about why children residing in certain parts of Central, Bono East and Upper East regions were at highest risk while their counterparts in parts of Greater Accra, Eastern, Northern, Volta, Upper West, Savannah, and Ashanti regions were at lower risk, further research in the form of qualitative studies in addition to consideration and examination of further potential predictors left out in this study is warranted. The study further recommend that the DHS survey program managers and implementers consider increasing the number of clusters to be used in future surveys, and to include all districts in Ghana to improve the level of precision of the model estimates and the spatial predictions.

## Supplementary Information

**Additional file 1.** Building the mesh, SPDE and projector matrices for estimation and prediction

**Additional file 2. **Figures for supplementary material for online interactive web-based maps for Figs. 4, 5, 6

## Data Availability

The datasets generated and/or analysed during the current study are available from the Measure DHS Program website http://dhsprogram.com/data/available-datasets.cfm.

## Abbreviations

CHPS: Community-based Health Planning and Services
DHS: Demographic and Health Surveys
EAs: Enumeration areas
GMIS: Ghana Malaria Indicator Survey
GSS: Ghana Statistical Service
ITNs: Insecticide-treated nets
INLA: Integrated Nested Laplace Approximation
MICS: Multiple Indicator Cluster Surveys
PMI: President’s Malaria Initiative
RDT: Rapid diagnostic test
SPDE: Stochastic Partial Differential Equation
SSA: Sub-Saharan Africa
SDG: Sustainable Development Goals
U5M: Under-five mortality
WAIC: Watanabe-Akaike information criterion
