# Supplementary material for: Mapping under-five child malaria risk that accounts for environmental and climatic factors to aid malaria preventive and control efforts in Ghana: Bayesian geospatial and interactive web-based mapping methods
Source: Malar J. 2022 Dec 15;21:384. doi: 10.1186/s12936-022-04409-x (PMC9756577; doi:10.1186/s12936-022-04409-x)
Supplement: Supplementary file 1 — Additional file 1. Building the mesh, SPDE and projector matrices for estimation and prediction [file 12936_2022_4409_MOESM1_ESM.docx]

**Additional file 1: Building the mesh, SPDE and projector matrices for estimation and prediction**

The data in this study is a point data which do not have explicit neighbours unlike an areal data. It will therefore be difficult to estimate the autocorrelation structure between points existing in our region of study. Thus, the study area was discretize to create a mesh to obtain set of neighbours to allow estimation of these autocorrelations between the points. The construction of the mesh in our study is critical because we used the SPDE strategy[1] to approximate the continuous Gaussian field via Gaussian Markov random field (GMRF). In this study, a triangulated mesh was created using the function inla.mesh.2d() from the R-INLA package. The study presented detailed description of mesh creation, SPDE and projector matrices in supplementary material S1. Detailed discussion about the procedures presented here are published elsewhere.[2]

The study used location coordinates as the initial mesh vertices and set the minimum allowed distance between points as 0.01 to avoid creating many small triangles where there are some very close points. The study also set the maximum allowed triangle edge lengths in the study region and in the extension as 0.3 and 5, respectively, to use small tringles within the study region and larger triangles in the extension. The study then build the SPDE model on the created mesh using inla.spde2.matern() function in the R-INLA package and set α which relates to the smoothness parameter of the process (i.e., α = ν + d/2) to be α=2 by setting the smoothness parameter ν = 1 and d=2. This was followed by the creation of the index set for the SPDE model using inla.spde.make.index() function by specifying the name of the spatial effect and the number of vertices in the SPDE and then created a projector matrix to project the spatially continuous Gaussian random field at the mesh nodes using the inla.spde.make.A() function by passing the mesh and the coordinates.

Furthermore, the study intends to predict under-five malaria prevalence as a continuous phenomenon over Ghana for both sampled and unsampled locations. To achieve this, the locations to predict the malaria prevalence were identified by setting the prediction locations to the locations of our covariate raster data which contained covariates used in the spatial model. The rasterToPoints() function from the raster package was used to extract the coordinates of the raster, and stored the coordinates and the values of the raster without missing observations. The study also created the matrix that projects the locations where the predictions will be done using inla.spde.make.A() function. Finally, the data was prepared for estimation and prediction by using the function inla.stack() to organize the data, effects (fixed and random) and the projector matrices. Detailed discussion about the procedures presented here are available elsewhere.[2]

**References**

1. Lindgren F, Rue H, Lindström J. An explicit link between Gaussian fields and Gaussian Markov random fields: the stochastic partial differential equation approach. J R Stat Soc. 2011;73:423-98.

2. Lindgren F, Rue H. Bayesian Spatial Modelling with R-INLA. J Stat Softw. 2015;63:1-25.
