# Supplementary material for: Mapping under-five child malaria risk that accounts for environmental and climatic factors to aid malaria preventive and control efforts in Ghana: Bayesian geospatial and interactive web-based mapping methods
Source: Malar J. 2022 Dec 15;21:384. doi: 10.1186/s12936-022-04409-x (PMC9756577; doi:10.1186/s12936-022-04409-x)
Supplement: Supplementary file 2 — Additional file 2. Figures for supplementary material for online interactive web-based maps for Figs. 4, 5, 6 [file 12936_2022_4409_MOESM2_ESM.zip › New folder/Additional file 2.docx]

**Additional file 2: Figures for supplementary material for online interactive web-based maps for Figures 4-6.**

Figure S1. Predicted malaria prevalence in 2019 among under-five children in Ghana. Interactive version of Figure 4.

Figure S2. SEs of predicted malaria prevalence in 2019 among under-five children in Ghana. Interactive version of Figure 5.

Figure S3. Predicted width of the 95% credible intervals of malaria prevalence in 2019 among under-five children in Ghana. Interactive version of Figure 6.
